# Therapeutic Potential of Annexins in Sepsis and COVID-19

**DOI:** 10.3389/fphar.2021.735472

**Published:** 2021-09-09

**Authors:** Louise Mui, Claudio M. Martin, Brent J. Tschirhart, Qingping Feng

**Affiliations:** ^1^ Division of Critical Care, Department of Medicine, Schulich School of Dentistry and Medicine, Western University, London, ON, Canada; ^2^ Lawson Health Research Institute, London Health Sciences Centre, London, ON, Canada; ^3^ Department of Physiology and Pharmacology, Schulich School of Dentistry and Medicine, Western University, London, ON, Canada

**Keywords:** sepsis, COVID-19, annexin (A1, A2 and A5), inflammation, coagulation

## Abstract

Sepsis is a continuing problem in modern healthcare, with a relatively high prevalence, and a significant mortality rate worldwide. Currently, no specific anti-sepsis treatment exists despite decades of research on developing potential therapies. Annexins are molecules that show efficacy in preclinical models of sepsis but have not been investigated as a potential therapy in patients with sepsis. Human annexins play important roles in cell membrane dynamics, as well as mediation of systemic effects. Most notably, annexins are highly involved in anti-inflammatory processes, adaptive immunity, modulation of coagulation and fibrinolysis, as well as protective shielding of cells from phagocytosis. These discoveries led to the development of analogous peptides which mimic their physiological function, and investigation into the potential of using the annexins and their analogous peptides as therapeutic agents in conditions where inflammation and coagulation play a large role in the pathophysiology. In numerous studies, treatment with recombinant human annexins and annexin analogue peptides have consistently found positive outcomes in animal models of sepsis, myocardial infarction, and ischemia reperfusion injury. Annexins A1 and A5 improve organ function and reduce mortality in animal sepsis models, inhibit inflammatory processes, reduce inflammatory mediator release, and protect against ischemic injury. The mechanisms of action and demonstrated efficacy of annexins in animal models support development of annexins and their analogues for the treatment of sepsis. The effects of annexin A5 on inflammation and platelet activation may be particularly beneficial in disease caused by SARS-CoV-2 infection. Safety and efficacy of recombinant human annexin A5 are currently being studied in clinical trials in sepsis and severe COVID-19 patients.

## Introduction

Sepsis is defined as life-threatening organ dysfunction caused by a dysregulated host response to infection (Singer et al., 2016). The Global Burden of Disease Study reported there were 11 million sepsis-related deaths globally (1 in 5 of all deaths) in 2017 (Rudd et al., 2020). Subsequently, coronavirus disease 2019 (COVID-19) caused by severe acute respiratory syndrome coronavirus-2 (SARS-CoV-2), a novel coronavirus (Wiersinga et al., 2020), has emerged as a cause of sepsis. While most people infected with this coronavirus are asymptomatic or only show mild symptoms, many have developed severe disease that requires hospitalization, of whom about 20–25% with critical COVID-19 have been admitted to intensive care units (ICU) (CanadaA of (2021) Epid, 2021). As of August 28, 2021, over 4.5 million deaths (2.1% of total cases) have been attributed to COVID-19 although this rate varies widely among countries, ranging from 0.8% in New Zealand to 7.8% in Mexico (www.worldometers.info/coronavirus).

Despite much research, no specific anti-sepsis treatment exists, and management relies on early administration of broad-spectrum antibiotics, resuscitative measures, and corticosteroids to reduce patient mortality (Rice and Bernard, 2005; Ferrer et al., 2009; Cohen et al., 2015). A number of therapeutics have been investigated for their potential as sepsis treatment, including drotrecogin alfa (activated human protein C), which briefly became known as the only drug approved specifically for use in sepsis before it was withdrawn from the market (Angus, 2012). Annexin A5 is a 36-kDa endogenous protein and part of a 12-member family of ubiquitously and constitutively expressed proteins, and possesses anticoagulant, anti-inflammatory and anti-apoptotic properties. The anticoagulant effect of annexin A5 is achieved through binding to phosphatidylserine (PS) on cell membranes of activated platelets and endothelial cells to prevent the assembly of prothrombinase complex and thrombin generation (van Genderen et al., 2008a; Gu et al., 2015a). Unlike activated protein C (Bernard et al., 2001), it does not have thrombolytic action and thus is less likely to cause adverse bleeding events. Additionally, annexin A5 improves nitric oxide signalling and vascular endothelial function through inhibition of inflammation and endothelial activation (Ewing et al., 2011a). These properties make it a promising therapeutic agent to reduce the consequences of endothelial injury in sepsis and COVID-19. In addition, annexin A1 and A2 have also been shown to have protective roles in coagulopathy and inflammation. Although other annexins such as annexin A3, which has been shown to mediate pathogen clearance and its expression is increased in neutrophils in patients with sepsis, their therapeutic potential in sepsis is currently not clear (Toufiq et al. 2020). In this review, we summarize existing knowledge of annexin functions and potential therapeutic applications of annexin A1, A2 and A5 in sepsis and COVID-19.

## Normal Functions of Extracellular Annexins

The annexin superfamily contains over 1,000 proteins found in over 65 different species of plants and animals (Gerke and Moss, 2002; Moss and Morgan, 2004). All annexins share a similar structural core that is highly conserved throughout all members of the superfamily. This core consists of four homologous domains of approximately 70 amino acid residues (except for Annexin A6 which contains eight domains) (Gerke and Moss, 2002), and gives annexins a common 3D structure.

Twelve annexin genes have been found in humans, dispersed among several chromosomes (Moss and Morgan, 2004). Though known primarily as an intracellular protein, several annexins are found extracellularly where they bind to various extracellular and cell membrane ligands and receptors that mediate systemic effects, most notably in coagulation and inflammation (Raynal and Pollard, 1994; Gerke and Moss, 2002; Lizarbe et al., 2013; Schloer et al., 2018). Key to a number of these interactions is the annexin’s conserved ability to bind with negatively charged phospholipids such as PS present on cell membrane surfaces (Schloer et al., 2018).

### Annexin A1

Annexin A1, also known as lipocortin, is found in a number of tissues, including lung, kidney, bone marrow, intestine, spleen, thymus, brain, and seminal fluid (Fava et al., 1989). Its extracellular activity has been studied extensively, specifically in the realm of inflammation (Fava et al., 1989). Annexin A1 must be externalized to exhibit its anti-inflammatory effects (Blume et al., 2009; Gavins and Hickey, 2012). Its release is regulated by glucocorticoids, and it inhibits the action of phospholipase A2, which subsequently blocks release of arachidonic acid, resulting in anti-inflammatory actions (Sheikh and Solito, 2018).

The key anti-inflammatory effect of extracellular annexin A1 is its interference and modification of the adhesion and migration of leukocytes, which are fundamental steps in the inflammatory response (Gavins and Hickey, 2012). Annexin A1 induces L-selectin shedding by neutrophils, likely by binding to formyl peptide receptors, which results in detachment of adherent leukocytes from endothelium, preventing their *trans*-endothelial migration (De Coupade et al., 2003; Gavins et al., 2003; Gavins and Hickey, 2012). Annexin A1 also binds and activates cell messengers that activate apoptosis machinery (Solito et al., 2003; Gavins and Hickey, 2012), which prevents necrosis and release of inflammatory factors. It has a role in controlling release of nitric oxide and inhibits cyclooxygenase 2 expression, thereby upregulating potent anti-inflammatory cytokines such as interleukin (IL)-10 (Minghetti et al., 1999). A recent study shows a role for annexin A1 in the regulation of nucleotide-binding oligomerization (NOD-), LRR- and pyrin domain-containing protein (NLRP3) inflammasome activation (Sanches et al., 2020a).

Annexin A1 also has a role in adaptive immunity. Exposing T cells stimulated with CD3 and CD28 to annexin A1 prolongs stimulation of AKT and extracellular signal-regulated kinase pathways, and increases proliferation of T cells (D’Acquisto et al., 2007; Sheikh and Solito, 2018). In tuberculosis, the absence of annexin A1 resulted in impaired CD8^+^ T cell response, and reduced dendritic cell mediated efferocytosis (Tzelepis et al., 2015). Additionally, annexin A1 promotes wound healing, as annexin A1 supplementation rescued defects in mice in intestinal wound repair caused by *NOX1* gene deficiency (Babbin et al., 2008; Leoni et al., 2013).

### Annexin A2

Annexin A2, also known as chromobindin-8, lipocortin II, and placental anticoagulant protein, is present in many cells, including endothelial cells, monocytes, macrophages, dendritic cells, trophoblast cells, and epithelial cells (Luo and Hajjar, 2013). Though extracellular activity has been observed, it works primarily while bound on the external leaflets of cell membranes, and is not appreciably found in plasma (Hajjar and Krishnan, 1999). Expressed as a tetramer with the S100A10 calcium binding protein, annexin II tetramer (AIIt) translocation to the cell surface is a key step in regulation of fibrinolysis (Hajjar and Krishnan, 1999), and serves as a co-receptor for plasminogen and tissue plasminogen activator (tPA) (Hajjar and Krishnan, 1999).

On the endothelial cell surface, AIIt promotes production of plasmin, resulting in the subsequent breakdown of fibrin (Hajjar and Krishnan, 1999). Annexin A2 deficient mice display deposition of fibrin in the microvasculature and incomplete clearance of injury-induced arterial thrombi (Ling et al., 2004). In humans, an increased annexin A2 expression in acute promyelocytic leukemia (APL) is associated with hyperfibrinolysis and bleeding (Menell et al., 1999; Liu et al. 2011) while reduced annexin A2 expression impairs cell surface fibrinolysis and may constitute a risk factor for venous thromboembolism (Fassel et al., 2021).

AIIt’s action on fibrinolysis regulation is both inhibitory and stimulatory. In the presence of tPA, it stimulates the production of plasmin, enhancing fibrinolysis. However, once plasmin is produced, AIIt rapidly degrades it by stimulating autoproteolysis, thereby inhibiting fibrinolysis (Fitzpatrick et al., 2000). The relationship between annexin A2, AIIt, and fibrinolysis is thus complex.

In sepsis and inflammation, annexin A2 has different roles at different stages of the disease process. In early stages, it limits vascular permeability, preventing edema and extravasation and thereby modulating leukocyte recruitment and inflammatory mediator release. Within hours, it protects internal membranes, preventing inflammasome actions by protecting lysosome membranes and preventing cytokine production and release. By late stages it promotes angiogenesis and wound healing (Dallacasagrande and Hajjar, 2020). However, annexin A2 can serve as a site of adhesion and entry for bacteria such as *Pseudomonas aeruginosa*, *Salmonella typhimurium* and *rickettsia* resulting in pro-inflammatory actions by causing apoptosis and release of inflammatory cytokines (Kirschnek et al., 2005; Miyahara et al., 2009; Jolly et al., 2014; He et al., 2019), may enable viral infection and replication (Taylor et al., 2018), and excessive angiogenesis induced by annexin A2 may induce tissue damage (Liu and Hajjar, 2016). Studies in sepsis models have found that knockout mice that lack annexin A2 have more severe disease and decreased survival in polymicrobial sepsis, with significantly higher bacterial load, worse tissue integrity, and greater distant organ damage (He et al., 2016). In humans, lower levels of annexin A2 correlated with increased sepsis severity (West et al., 2015), and plasmin generation capacity is greatly reduced in septic patients compared to healthy controls (Lanfranco et al., 2013).

### Annexin A5

Annexin A5 inhibits blood coagulation. Annexin A5 is found in human placenta (syncytiotrophoblasts), blood vessels throughout the body, and membranes in the heart, lung, and liver. Annexin A5 is an accessory protein that helps direct cytosolic phospholipase A2 to the membrane of activated platelets where it controls its activity (Tzima et al., 2000; Tzima and Walker, 2000) and is highly expressed in cell types including vascular endothelial cells that serve a barrier function (Flaherty et al., 1990; Wen et al., 1999; Rand et al., 2012a). The protein self-assembles into trimers that form a protective 2-dimensional crystal lattice when it binds to PS exposed on cell membranes, in the presence of calcium (Reutelingsperger and Van Heerde, 1997; van Genderen et al., 2008b). This lattice acts as a shield, as annexin A5 competes with coagulation factors and prothrombin from binding to PS, and thereby prevents formation of the prothrombin complex and thrombin, resulting in an anticoagulation effect (Andree et al., 1992; Van Heerde et al., 1994; Reutelingsperger and Van Heerde, 1997; van Genderen et al., 2008b).

Annexin A5 binds the apical surface of syncytiotrophoblasts, and maintains normal blood flow through the placenta by preventing coagulation (Krikun et al., 1994). Its shielding ability also promotes membrane resealing in human trophoblasts in pregnancy (Krikun et al., 1994; Rand et al., 1997). Disruption of this shield in pregnant women with antiphospholipid syndrome may cause miscarriage, as the apical surface of the placenta becomes thrombogenic (Rand et al., 1997; Hunt et al., 2011).

Protein kinase C (PKC) is a family of protein kinase enzymes involved in a variety of signal transduction pathways such as apoptosis, cell proliferation, differentiation, and T cell activation through phosphorylation of serine and threonine amino acid residues on the target proteins. PKCs are activated by increases in intracellular diacylglycerol (DAG). There are 15 PKC isozymes in humans. PKC-α, -*β*, -*γ*, -*δ*, and -*ε* isoforms have important roles in atherosclerosis, myocardial ischemia/reperfusion injury, cardiac hypertrophy, fibrosis and heart failure (Singh et al., 2017). Annexin A5 has been shown to transiently interact with PKC-δ in cells after PKC-δ stimulation, but before PKC-δ translocates to the particulate fraction (Kheifets et al., 2006). In addition, the recruitment of PKC-θ to the membrane, NF-κB signaling and T-cell activation are impaired in ANXA5 knockout T cells, suggesting a critical role of annexin A5 in T-cell activation (Hu et al., 2020).

Annexin A5 protects cells from apoptosis and reduces inflammation. PS on cell membranes is also a key signal molecule in apoptosis, signaling for immune cells to phagocytose apoptotic cells (Balasubramanian and Schroit, 2003). Annexin A5’s shielding ability inhibits this phagocytosis by physically shielding surface expression ligands located near PS residues, as well as down-regulating the expression of those ligands, and may play a role in preventing the engulfment of living cells expressing PS (Kenis et al., 2006). Notably, annexin A5 inhibits proteolytic activation of caspase-3, an executioner caspase critical to apoptosis (Gidon-Jeangirard et al., 1999). Annexin A5 also bind to PS on microparticles released from platelets and leukocytes. The microparticles have pro-coagulant and pro-inflammatory properties, and are implicated in a number of disease conditions, including atherosclerosis, cancer, autoimmune disease, and sepsis (Ardoin et al., 2007). Not only is the annexin A5 binding and shielding capable of acting as a physical constraint to blebbing and microparticle shedding, its binding to microparticle membrane is sufficient to reduce coagulant and inflammatory processes promoted by these microparticles (Thiagarajan and Tait, 1991). Furthermore, annexin A5 coated microparticles or extracellular vesicles (EVs) are more readily taken up by monocytes and macrophages. In mice infected with *E. coli*, annexin A5 treatment decreases circulating EVs and dose-dependently delays the development of sepsis (Tontanahal et al., 2021). Additionally, annexin A5 reduces inflammation by shifting the macrophages from the classically activated phenotype (M1) to alternatively activated (M2) phenotype via directly interacting with pyruvate kinase M2 (PKM2) in the liver (Xu et al., 2020).

Annexin A5 has additional actions that may be important in health and disease. It binds to glycosaminoglycans such as heparin and heparan sulfate, a major component of cell surface proteoglycans of endothelial cells (Capila et al., 2001), promoting anticoagulation. Annexin A5 interacts with the N-terminal leucine-rich repeats (LRR) of polycystin-1, a transmembrane protein expressed apical membranes, adherens junctions and desmosomes. Annexin A5 inhibits the effects of polycystin-1 on the recruitment of E-cadherin to reform junctions in canine kidney cells (Markoff et al., 2007). Annexin A5 may be involved in regulation of vascular endothelial cell proliferation as a signaling protein for vascular endothelial growth factor receptor-2 (VEGFR-2) by directly interacting with the intracellular domain of the receptor (Wen et al., 1999). Annexin A5 also binds to distinct sites of negatively charged phospholipids present in oxidized low-density lipoprotein (Van Tits et al., 2005), and has been shown to reduce vascular inflammation and improve endothelial function in ApoE knockout mice of atherosclerosis (Ewing et al., 2011b).

## Therapeutic Applications of Annexins and Analogue Molecules

### Current Clinical Applications

Annexin A5 is the only annexin currently used in clinical practice. Based on the observation that PS is expressed on the surface of mammalian apoptotic cells (Fadok et al., 1992), which annexin A5 binds with a high affinity, annexin A5 can be used in flow cytometry to detect apoptotic cells after chemotherapy (Koopman et al., 1994; Belhocine et al., 2002), cord blood to improve prediction of potency for engraftment (Duggleby et al., 2012), and detection of anti-platelet antibodies for the diagnosis of antiphospholipid syndrome (Tomer et al., 2007). It has been evaluated as a marker for lymphocyte apoptosis in sepsis but results changed with storage time prior to the assay (Greineder et al., 2007). Annexin A5 can also be used to quality check stored platelets, as they expose more PS residues over time (Tait et al., 1999).

Various annexin A5 imaging probes have been found to be safe in human imaging studies *in vivo* (Boersma et al., 2005), and have been used to image apoptosis in various conditions, including cardiovascular disease (Thimister et al., 2003; Korngold et al., 2008), allograft rejection in transplants (Blankenberg et al., 2000; Kown et al., 2001; Narula et al., 2001), evaluation of cancer treatment efficacy by assessing apoptosis of tumor cells after treatment (Belhocine et al., 2002; Mochizuki et al., 2003; Kartachova et al., 2004).

### Potential Therapeutic Applications

#### Annexin A1

Difficulty in manufacturing large quantities of the annexin A1 protein, and antibody formation against injected protein have limited its therapeutic use (Flower and Rothwell, 1994). Because of this, small peptides derived from the N terminal region of annexin A1 were developed to retain its biological activity. Of the three analogue peptides (Gavins and Hickey, 2012), Ac2-26 has undergone the most extensive evaluation but only in animal models (Table 1).

**TABLE 1:** Effects of Annexin A1 and analogue peptide treatment in experimental studies.

Annexin or analogue	Cell/organ of interest	Effect	Research model	Reference
AC2-26	Cardiomyocytes and heart	Inhibition of sepsis-induced cardiomyocyte apoptosis	LPS-induced endotoxemia in rats	Zhang et al. (2018)
AC2-26	Brain	Reduced leukocyte adhesion and resultant anti-inflammatory effects, inhibition of prostanoid and NO release	Endotoxin-induced inflammation in mice	Gavins et al. (2012)
AC2-26	Lung	Retain leukocytes in vessels and decrease leukocyte migration with resultant anti-inflammatory effects, reduce inflammatory cytokine release	Endotoxin-induced inflammation in mice	da Cunha et al. (2012)
AC2-26	Lung	Preservation of pulmonary parenchyma and reduced inflammatory cells, reduced leukocyte influx, attenuation of lung edema, apoptosis and tissue damage	COPD in rats	Possebon et al. (2018)
Recombinant annexin A1	Cardiomyocytes and heart	Dose-dependent reduction of tissue damage, reduction of extravasated leukocytes, inhibition of inflammatory cytokine release	Myocardial ischemia-reperfusion in rats	D’Amico et al. (2000)
AC2-26	Cardiomyocytes and heart	Inhibition of leukocyte migration and recruitment, reduction in myeloperoxidase activity in affected myocardium, effective against IR injury up to 60 min after reperfusion	Myocardial ischemia-reperfusion in rats	La et al. (2001)
AC2-26	Cerebral microvasculature	Reduced leukocyte adhesion, inhibited reduction of neurological score	Cerebral ischemia-reperfusion in mice	Gavins et al. (2007)
AC2-26	Kidney	Preservation of renal function and structure, protection of GFR and tubular function, prevention of neutrophil extravasation, anti-inflammatory actions	Renal ischemia-reperfusion in rats	Facio et al. (2011)
CR-Ac_2-50_	Heart	Attenuation of myocardial dysfunction	Cecal ligation and perforation in mice	Gobbetti et al. (2014)

Annexin A1 and its derivative peptides have been shown to have therapeutic effect in various disease states, including sepsis (Gavins et al., 2012; Zhang et al., 2018), lung inflammation (da Cunha et al., 2012) and chronic obstructive pulmonary disease (COPD) (Possebon et al., 2018), myocardial infarction (D’ Amico et al., 2000; La et al., 2001; Qin et al., 2015), and intestinal wound repair (Babbin et al., 2008). Studies confirmed that endogenous annexin A1 plays a role in promoting phagocytosis (Yona et al., 2006; Scannell et al., 2007), and its derived peptide Ac2-26 increases macrophage phagocytosis of apoptotic polymorphonuclear leukocytes (PMNs) (Maderna et al., 2005). Ac2-26 administration in murine lipopolysaccharide (LPS) models of sepsis has demonstrated inhibition of cardiomyocyte apoptosis and myocardial damage (Zhang et al., 2018), reduced leukocyte adhesion and cerebrovascular inflammatory response in the brain (Gavins et al., 2012), and reduced inflammatory cytokine release and subsequent decreased inflammation in lung (da Cunha et al., 2012; Possebon et al., 2018). Another annexin A1 analogue (CR-Ac_2-50_) reduces inflammation and attenuates myocardial dysfunction in a polymicrobial sepsis model (Dalli et al., 2013; Gobbetti et al., 2014).

In ischemic insult, Ac2-26 and annexin A1 demonstrate cardioprotective effects in rats and mice, reducing infarct size and myeloperoxidase activity (D’ Amico et al., 2000; La et al., 2001; Qin et al., 2015), although not as completely as corticosteroid treatment (Ritchie et al., 2003). In mice models of ischemia and reperfusion, annexin A1 mimetics prevent white blood cell adhesion and markers of inflammation in cerebral infarct (Gavins et al., 2007) and inhibit macrophage infiltration, maintaining glomerular filtration rate and urine osmolality while preventing acute tubular necrosis in renal ischemia (Facio et al., 2011).

#### Annexin A2

Zhang and co-workers reported that annexin A2 and its tetramer AnxA2-S100A10 directly activated human macrophages through toll-like receptor-4 (TLR4) signaling, facilitating its internalization in the cell and signaling for release of anti-inflammatory cytokines (Zhang et al., 2015). However, annexin A2 has not been explored as a therapeutic. This may be due to anticoagulant and anti-inflammatory properties that are weaker than annexin A1 or A5, mechanisms of action for modulation of fibrinolysis that are not fully understood and variable based on tissue type as shown with its pleiotropic activity (Bharadwaj et al., 2013), and potential pro-inflammatory actions that facilitate bacterial and viral infections (Taylor et al., 2018; He et al., 2019). Additionally, increased annexin A2 expression is a common phenomenon in many cancers, and correlates to adverse clinical outcomes in patients (Christensen et al., 2018). So, while annexin A2 has endogenous anticoagulant activity, it may serve as a potential therapeutic target rather than a treatment.

#### Annexin A5

Because of its high affinity binding to PS exposed on the membrane of apoptotic cells, various annexin A5 imaging probes have been used and found to be safe in human imaging studies (Boersma et al., 2005), including cardiovascular disease (Thimister et al., 2003; Korngold et al., 2008), allograft rejection in transplants (Blankenberg et al., 2000; Kown et al., 2001; Narula et al., 2001), and evaluation of cancer treatment efficacy by assessing apoptosis of tumor cells after treatment (Belhocine et al., 2002; Mochizuki et al., 2003; Kartachova et al., 2004). Annexin A5 and its human recombinant homodimer, diannexin, which was designed to have a longer circulating half time and greater binding affinity (Rand et al., 2012b), has been explored as a therapeutic protein in a wide range of disease conditions and contexts, most notably inflammation, sepsis, hemorrhage, coagulopathy, ischemia, and organ transplant. However, most of these studies are in animal models of endotoxemia (Table 2).

**TABLE 2:** Effects of Annexin A5 and analogue peptide treatment in experimental and clinical studies.

Annexin or analogue	Tissue/organ of interest	Effect	Research model	Reference
Diannexin	Platelets	Binds PS-exposing platelets, attenuates thrombin generation and fibrin clot formation, increases tail bleeding time in high doses in mice	Human cells *in vitro*, mouse model *in vivo*	Rand et al. (2012)
Annexin A5	Macrophages	Binds with high affinity to LPS on Gram-negative bacteria, neutralizes LPS activity, reduces macrophage response to LPS, reduces endotoxin effect of LPS in mice	Mouse macrophage *in vitro*, LPS-induced endotoxemia in mice	Rand et al. (2012)
Annexin A5	Immune cells, systemic	Increases survival rate of LPS-induced endotoxemia and CCI-induced sepsis in mice, inhibits LPS-induced activation and maturation of dendritic cells and reduces cytokine production, inhibiting TLR4 signalling, reduces pro-coagulation in septic conditions	LPS-induced endotoxemia and cecal content injection (CCI)-induced sepsis in mice	Park et al. (2016)
Human recombinant annexin A5	Cardiomyocytes and heart	Repairs LPS-induced adherens junction disruption, improves hemodynamics and prevents cardiac inflammation in endotoxemia	LPS-induced endotoxemia in mice	Gu et al. (2015)
Human recombinant annexin A5	Cardiomyocytes and heart	Inhibits MAPK, Akt, and NK-κB activation in endotoxemia; inhibits TNF-α and IL-1β expression in endotoxemia, improves cardiac function in endotoxemia, improves animal survival in endotoxemia, inhibits LPS-binding to TLR4/MD-2 fusion protein	LPS-induced endotoxemia in mice	Arnold et al. (2014)
Diannexin	Lung, kidney, and gut	Infusion before hemorrhage decreased renal and gut dysfunction, decreased lysophosphatidic acid levels and subsequently reduces vascular permeability and platelet aggregation	Hemorrhage and resuscitation in rats	Beattie et al. (2019)
Annexin A5	Monocytes	Inhibits LPS-induced procoagulant activity on human monocytes dose dependently	Human cells *in vitro*	Sato et al. (2004)
Recombinant rat annexin A5	Perivascular cells	Anxa5 null perivascular cells demonstrate defect in membrane repair, extracellular addition of anxA5 promotes membrane repair, anxA5 binds exclusively to disrupted membrane area	Mouse cells *in vitro*	Bouter et al. (2011)
Diannexin	Liver	Prevents liver damage, protects against surface injury to hepatic sinusoidal endothelial cells (SECs), abolishes inflammatory cell recruitment, inhibits aggregation and vascular adherence of platelets, restores sinusoidal blood flow	Ischemia-reperfusion injury (IRI) in mice	Teoh et al. (2007)
Diannexin	Liver	Protects against cold IRI, depresses chemokine/adhesion molecule expression and endothelial activation, decreases leukocyte traffic, reduces pro-inflammatory expression, depresses apoptosis and upregulates cytoprotective molecules	cold ischemia-reperfusion injury in rats	Shen et al. (2007)
Diannexin	Lung	improves function of transplanted graft, reduced alveolar fibrin deposition, suppresses cell death, suppresses intragraft proinflammatory cytokine expression, reduces fibrinolysis	Lung transplant and ischemia-reperfusion injury in rats	Hashimoto et al. (2016)
Diannexin	Kidney	Reduced tubule damage and leukocyte influx after renal IRI and improved renal function	Renal ischemia-reperfusion injury in mice	Wever et al. (2011)
Diannexin	Assess safety and tolerability	Improved post-transplant 24 h urine output, 29 days eGFR values, and was associated with less need and fewer days on dialysis compared to control	Phase 2 clinical trial, human marginal donor kidneys	Cooper et al. (2010); NCT00615966
Diannexin	Assess safety, efficacy and pharmacokinetics	Terminated after recruiting 21/591 patients	Phase 2/3 trial in kidney transplant recipients	Tsapepas et al. (2013); NCT01442337
Recombinant human annexin A5 (SY-005)	Evaluate safety, tolerance and pharmacokinetics	SY-005 was well tolerated with no major safety concerns	Phase 1 clinical trial, healthy subjects	NCT04217629
Recombinant human annexin A5 (SY-005)	Assess safety, feasibility and pharmacokinetics	Recruiting	Phase 2 clinical trial, COVID-19 patients with sepsis	NCT04748757
Recombinant human annexin A5 (SY-005)	Evaluate safety, tolerability and pharmacokinetics	Not yet recruiting	Phase 2 clinical trial in sepsis	NCT04898322
Recombinant human annexin A5	Evaluate safety, tolerability and pharmacokinetics	Recruiting	Phase 1 clinical trial in healthy subjects	NCT04850339

Annexin A5 can bind LPS directly to reduce its activity and macrophage response to LPS, and pretreatment with annexin A5 in mice reduces serum tumor necrosis factor *α* (TNF-α) concentrations similar to control animals (Rand et al., 2012a). Annexin A5 blocks TLR4 signaling in dendritic cells and cardiomyocytes, thereby reducing LPS induced proinflammatory cytokine release of IL-1β, IL-6 and TNF-α (Arnold et al., 2014; Park et al., 2016). It also inhibits late acting mediators of systemic inflammation such as high mobility group box 1 (HMGB-1), inducing an anti-inflammatory response (Park et al., 2016). Annexin A5 can repair LPS induced damage to cardiomyocyte adherens junctions, preventing cardiac inflammation, and improving hemodynamics in mice (Gu et al., 2015b). Animal survival in endotoxemia was improved, showing significant improvements not only when annexin A5 was injected immediately after LPS injection, but also with delayed annexin A5 treatment 4 h after LPS injection. This therapeutic window could be relevant to clinical sepsis (Arnold et al., 2014). Besides beneficial results shown in heart tissue in endotoxemia, annexin A5 treatment also improved tissue damage and function in the liver (Park et al., 2016), and gut (Beattie et al., 2019) in similar conditions, with anti-inflammatory effects reducing endothelial damage, and protective effects against sepsis induced coagulopathy by preventing fibrin deposition in organ microvasculature (Beattie et al., 2019).

Annexin A5 and diannexin’s ability to bind PS with high affinity make them potent inhibitors of platelet activity by preventing thrombin generation and reducing platelet accumulation. In mice, diannexin treatment inhibited thrombi formation by reducing platelet accumulation, and increased blood loss after tail tip transection (Rand et al., 2012b). In human monocytes, annexin A5 inhibited procoagulant activity induced by LPS, and a relatively low concentration of annexin A5 (0.1 μg/ml) was sufficient for anticoagulant effects to occur with an IC_50_ of 0.6 μg/ml (or 17.6 nM), demonstrating its potency (Sato et al., 2004). Annexin A5 also reduced thrombin generation induced by cecal ligation and perforation, which is a clinically relevant model of sepsis (Wang et al., 2018).

Annexin A5 has also generated interest in ischemia-reperfusion injury and organ transplant, based on the anticoagulant and anti-inflammatory effects, as well as endogenous annexin A5’s ability to promote membrane repair in certain tissues (Bouter et al., 2011). In murine models of hepatic ischemia reperfusion injury, diannexin treatment preserved sinusoidal endothelial cell integrity, prevented cell swelling and inflammatory cell recruitment, and reduced inflammatory mediator release, thereby reducing hepatic apoptosis and restoring sinusoidal blood flow (Shen et al., 2007; Teoh et al., 2007). In a murine model of lung transplant, diannexin treatment produced protection from inflammation, cell death, and fibrinolysis, as well as improvement of compromised organ function (Hashimoto et al., 2016).

Therapeutic use of diannexin in humans is limited. In a phase 2 trial with 58 renal transplant patients receiving marginal donor kidneys, a single diannexin intravenous bolus of 400 μg/kg was associated with improved renal function and lesser need for dialysis compared to control patients (Cooper et al., 2010). However, a subsequent multicenter phase 2/3 trial evaluating the efficacy, safety, and tolerability of diannexin in kidney transplant recipients was terminated prematurely after recruiting 21 patients out of 591 planned (NCT01442337) based on additional review of pre-clinical toxicology data (Tsapepas et al., 2013).

A randomized and placebo-controlled phase 1 clinical trial was conducted to evaluate safety, tolerance and pharmacokinetics of recombinant human annexin A5 (SY-005) in 94 healthy subjects (NCT04217629). The trial was recently completed. Doses of intravenous injection of SY-005 from 0.75 to 20 mg per person were well tolerated without any major adverse events, suggesting that recombinant human annexin A5 is safe in healthy subjects. Another phase 1 study using annexin A5 as a therapeutic drug is recruiting healthy volunteers (NCT04850339).

## Potential Areas of Exploration for Use of Therapeutic Annexins and Their Analogues

**FIGURE 1:**
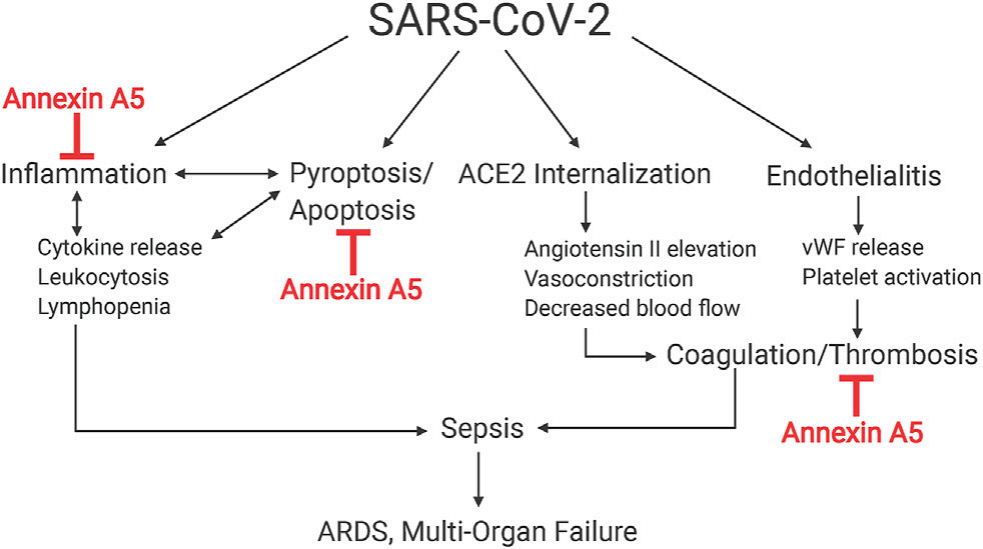
Effects of SARS-CoV-2 infection on host cells. SARS-CoV-2 exploits ACE2 receptors to gain entry to cells and causes endocytosis of ACE2 receptors. This internalization reduces availability of ACE2 in affected cells, causing systemic renin-angiotensin system deregulation and subsequent up-regulation of inflammatory responses, pyroptosis and release of inflammatory cytokines. Direct invasion of endothelial cells by SARS-CoV-2 and ensuing inflammation results in endotheliitis and increased release of von Willebrand factor from endothelial cells, promoting clotting in vessels.

The emergence of severe acute respiratory syndrome coronavirus-2 (SARS-CoV-2) on a global scale has driven a large need for treatments of COVID-19 induced sepsis and acute respiratory distress syndrome (ARDS). In COVID-19, SARS-CoV-2 enters the host cells *via* angiotensin converting enzyme 2 (ACE2) (Figure 1) and several proteases including transmembrane protease serine 2, furin and cathepsin L/B (Gheblawi et al., 2020; Hoffmann et al., 2020). Once inside the cell, it replicates rapidly and causes a systemic proinflammatory response with increased cytokine levels, leukocytosis, lymphopenia (in CD4^+^ and CD8^+^ T cells) and decreased interferon-*α* (IFNα) expression in CD4^+^ T cells. SARS-CoV-2 infiltration of cells is also capable of activating inflammasomes, such as NOD-, LRR- and pyrin domain-containing protein (NLRP3), which eventually leads to pore formation on the cell surface, IL-1β and IL-18 secretion and eventual pyroptosis, which further promotes inflammation (Yap et al., 2020). Thrombocytopenia, elevated prothrombin time, and high levels of D-dimers seen in COVID-19 infection suggest coagulopathy (Prompetchara et al., 2020), and though the initial target organ is the lung, the thromboinflammatory process subsequently can affect all organs of the body (Oudkerk et al., 2020). The imbalance between pro- and anti-inflammatory responses causes dysfunction of multiple organs, including the lung, liver, kidney, and the cardiovascular system, as well as coagulation/thrombolysis system, leading to septic shock with a high mortality (Blanco-Melo et al., 2020).

**FIGURE 2:**
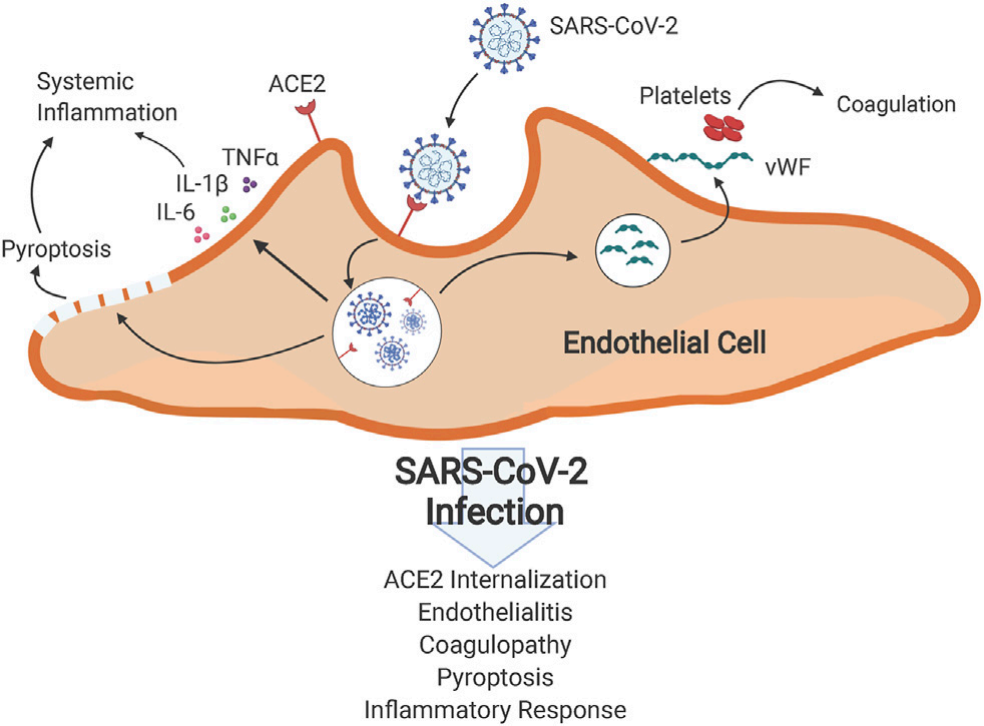
Schematic of hypothesis. Multifaceted actions of annexin A5 that are proposed to mitigate ARDS and multi-organ failure in coronavirus 2019 disease. SARS-CoV-2 causes sepsis and subsequent ARDS and multi-organ failure by multiple mechanisms, notably by promoting systemic inflammation and coagulopathy by its ability to internalize ACE2 and cause endotheliitis. Annexin A5 is hypothesized to impede these actions by its ability to reduce inflammation in disease states, inhibit thrombosis and lessen coagulopathy.

Annexins may have therapeutic potential in viral sepsis. While the mechanism of sepsis is different between bacterial and viral insults, they share commonalities in the activated receptors and released cytokines (DeMerle et al., 2021). To this end, annexins could be an effective therapy for patients suffering from COVID-19 induced sepsis or coagulopathy (Figure 2). Generally, severe cases of COVID-19 are associated with excessive cytokine release, with large amounts of IL-6, IL-1β, IL-2, IFN-γ, TNF-α, and other cytokines released (Fraser et al., 2020). IL-6 is a key inflammatory cytokine in SARS-CoV-2 infection, and its levels in COVID-19 patients are higher than those usually seen in severe bacterial sepsis (Levi, 2020; Paar et al., 2020). IL-6 levels in COVID-19 patients are a strong predictor of mortality and lung damage, and it has been suggested as a key therapeutic target in COVID-19 associated pathologies (Levi, 2020; Paar et al., 2020). Annexins have been found in various animal models to reduce the levels of IL-6, so there may be potential for its use in treatment of COVID-19 inflammation as an adjunct or alternative approach to anti-IL-6 receptor antibodies (Shen et al., 2007; Wever et al., 2011; Possebon et al., 2018). Additionally, NLRP3 activation promotes IL-1β expression and induces pyroptosis (Gao et al., 2018). A recent study showed that addition of Ac2-26 peptide decreases levels of IL-1β in macrophages, suggesting inhibition of the NLRP3 inflammasome (Sanches et al., 2020b). It is therefore possible that annexin A1, and possibly other annexins such as A5, may also be capable of protecting cells against SARS-CoV-2 induced pyroptosis.

COVID-19 systemic inflammation is accompanied by hemostatic abnormalities, especially in severe disease, with elevated coagulation, complement activation, higher incidence of venous thromboembolic events, and disseminated intravascular coagulopathy (DIC) (Levi et al., 2020; Paar et al., 2020). Heparin has been reported to reduce mortality due to SARS-CoV-2, and anticoagulation has been suggested as an important step to managing COVID-19 and mitigating the interplay between inflammation and thrombosis that occurs in the disease (Magro, 2020). Annexin A1 analogue Ac2-26 and recombinant human annexin A5, which have been shown in animal models of sepsis and other inflammatory models to reduce expression of pro-inflammatory cytokines, possess anticoagulant properties *via* inhibition of thrombin generation and platelet aggregation, and modulate the response to apoptosis, are promising therapeutic agents to reduce the severity of inflammatory processes in COVID-19. However, the exact mechanism of COVID-19 coagulopathy is not fully understood. Some studies of COVID-19 describe a pattern similar to DIC (Levi et al., 2020; Paar et al., 2020), which is seen in bacterial sepsis, while others suggest that COVID-19 associated coagulopathy is different from classic sepsis induced coagulopathy (SIC) and DIC. Direct endothelial infection by SARS-CoV-2 through ACE2, which is abundantly expressed on endothelial cells, induces endothelialitis and releases von Willebrand factor (vWF) and upregulates tissue factor expression, promoting thrombosis (Varga et al., 2020). Additionally, a function of ACE2 is to catalyze angiotensin II conversion to angiotensin-(1–7). SARS-CoV-2 binding to ACE2 and subsequent internalization downregulates surface ACE2 expression, leading to angiotensin II elevation, vasoconstriction, and decreased blood flow, which enhances thrombosis (Liu et al., 2020). Further, endothelial cell activation and glycocalyx degradation can stimulate platelet activation and microthrombi generation. Notably, we recently reported in critically ill COVID-19 patients evidence for greater endothelial cell activation and glycocalyx degradation than non-COVID-19 subjects (Fraser et al., 2020). It is possible that annexin A5 treatment could mitigate these effects and consequences of endothelial injury.

Extracellular HMGB-1 is another potential therapeutic target. Extracellular HMGB-1 is released from dying cells and forms complexes with extracellular DNA, RNA, and other molecules after lytic cell death, which are endocytosed. It has been proposed that SARS-CoV-2 RNA may enter cells via HMGB1-assisted transfer, promoting viral infection (Andersson et al., 2020a). Plasma levels of HMGB-1 in COVID-19 patients admitted to the ICU were significantly elevated in comparison to healthy subjects and non-ICU COVID-19 patients (Chen et al., 2020a). SARS-CoV-2 (Choudhury and Mukherjee, 2020) and other viruses, including RSV, Ebola, and dengue (Olejnik et al., 2018), also activate toll-like receptor-4 (TLR4) to induce the inflammatory response. High mobility group protein-box 1 (HMGB-1) is another ubiquitous protein implicated in the inflammatory response to infections, including SARS-CoV-2 (Sundén-Cullberg et al., 2005; Andersson et al., 2020b; Chen et al., 2020b). Since annexin A5 inhibits HMGB-1 interaction with TLR4 in models of sepsis and inflammation (Park et al., 2016), it could interfere with HMGB1-mediated SARS-CoV-2 infection.

We have started a randomized, double blind and placebo-controlled phase 2 clinical trial to evaluate safety, tolerance and pharmacokinetics of recombinant human annexin A5 in COVID-19 patients with sepsis (NCT04748757). The intervention is SY-005, 50 or 100 μg/kg intravenously every 12 h for 7 days, with a placebo comparator. The primary outcomes are feasibility metrics of enrollment, treatment delivered per protocol, data collection per protocol, and adverse events. At the time of this submission, 12 patients were recruited with no serious adverse events.

## Final Conclusion

Annexins are endogenously produced proteins with anti-inflammatory, anti-apoptotic, and anticoagulant activities. Annexins and their peptide analogues, when administered in various conditions in different animal models of disease, can alleviate and improve inflammation, coagulation, and ischemia-reperfusion injury. Evidence to date suggests that annexins could be used as a treatment, prophylactic therapy, or adjuvant therapy in various contexts, most notably sepsis, coagulopathy, and ischemic-reperfusion injury. Confirmation of these benefits is required in human trials. Annexin therapy could thus be an effective therapy in various disease conditions, especially sepsis, and novel diseases such as SARS-CoV-2 infection.

## Abbreviations

ACE2, angiotensin converting enzyme 2; AIIt, annexin II tetramer; APL, acute promyelocytic leukemia; ARDS, acute respiratory distress syndrome; DIC, disseminated intravascular coagulopathy; HMGB-1, high mobility group box 1; IFN, interferon; IL, interleukin; LPS, lipopolysaccharide; LRR, leucine-rich repeat; NLRP-3, NOD-, LRR- and pyrin domain-containing protein; PMN, polymorphonuclear leukocyte; PS, phosphatidylserine; SARS-CoV-2, severe acute respiratory syndrome coronavirus-2; TLR4, toll-like receptor-4; TNF-α, tumor necrosis factor *α*; tPA, tissue plasminogen activator; VEGFR-2, vascular endothelial growth factor receptor-2; vWF, von Willebrand factor.
